# What Defines a Host? Oviposition Behavior and Larval Performance of *Spodoptera frugiperda* (Lepidoptera: Noctuidae) on Five Putative Host Plants

**DOI:** 10.1093/jee/toac056

**Published:** 2022-12-14

**Authors:** Trevor M Volp, Myron P Zalucki, Michael J Furlong

**Affiliations:** School of Biological Sciences, The University of Queensland, St Lucia, Queensland, Australia; Department of Agriculture and Fisheries, Toowoomba, Queensland, Australia; School of Biological Sciences, The University of Queensland, St Lucia, Queensland, Australia; School of Biological Sciences, The University of Queensland, St Lucia, Queensland, Australia

**Keywords:** host plant specificity, polyphagy, host range, *Zea mays*, preference-performance hypothesis

## Abstract

When an invasive species first breaches quarantine and establishes in yet another country, it invariably causes consternation for growers, in part because of incomplete understanding of the plants that are at risk. The Fall Armyworm, *Spodoptera frugiperda* (J.E. Smith) is the most recent example in Australia. The number of plants that this polyphagous noctuid is reported to attack is vast, including many crop species. Consequently, initial reactions from grower industry groups that perceived themselves at risk were to demand emergency use of insecticides. Yet the field evidence suggests that many crops might not be at risk and since *S. frugiperda* arrived in Australia, maize crops have suffered most damage, followed by sorghum. We question the accuracy of some of the claims of reported host plants of *S. frugiperda* and report experiments that compared oviposition behavior, neonate silking behavior, and larval performance on five crops: the known hosts maize and sorghum, and the putative hosts cotton, peanut, and pigeon pea. Maize ranked highest in all preference and performance measures, followed by sorghum and peanut, with pigeon pea and cotton ranking lowest. Although *S. frugiperda* can survive, develop, and pupate on the crop species we examined, cotton and pigeon pea are not preferred by the pest in either the larval or adult stages. We suggest that before a plant is listed as a host for a given insect that the evidence should be fully reported and carefully evaluated. Collecting an immature insect from a plant does not make that plant a host!

The Fall Armyworm, *Spodoptera frugiperda* (J.E. Smith), has rapidly become a major pest of global agriculture. Initially restricted to the Americas, *S. frugiperda* was first reported in Africa in 2016 (Goergen et al. 2016). It has since spread through Africa (Day et al. 2017, Feldmann et al. 2019), Asia (Sharanabasappa et al. 2018, Wang et al. 2020), and Australia (Maino et al. 2021) within a 4-yr period. Throughout the invasion front, *S. frugiperda* has infested crops resulting in significant yield losses and consequent economic costs (Day et al. 2017, Yan et al. 2021). Due to the recent worldwide spread of *S. frugiperda*, the extent of its global economic impact remains unknown, but it is substantial.

*Spodoptera frugiperda* is considered a polyphagous species with a strong preference for plants in the Poaceae. A widely cited recent review listed 353 ‘host plants’ of *S. frugiperda* from 76 plant families, including many cultivated crops (Montezano et al. 2018). Of the host plant species listed by Montezano et al. (2018), 30% belong to the Poaceae. Despite this broad host list, the most regularly impacted crop on the invasion fronts in Africa, Asia, and now Australia appears to be maize (*Zea mays*; Day et al. 2017, Yan et al. 2021).

A strong oviposition preference of *S. frugiperda* for *Z. mays* has been demonstrated in the laboratory (Guo et al. 2021, He et al. 2021a, Sotelo-Cardona et al. 2021). This oviposition preference correlates with larval performance, as *Z. mays* appears to be highly suitable for *S. frugiperda* larval survival and development when compared with other crop species (Ali et al. 1990b, Guo et al. 2021, He et al. 2021b). There are two genetically distinct ‘strains’ of *S. frugiperda*: the ‘corn’ and ‘rice’ strains (Pashley et al. 1985, Nagoshi et al. 2007a); these are morphologically identical, but they differ in their host plant preferences (Meagher et al. 2011). The ‘corn’ strain prefers to oviposit on *Z. mays*, whereas the ‘rice’ strain prefers pasture grasses and rice for egg laying (Pashley et al. 1985, Meagher et al. 2011, Hafeez et al. 2021). Evidence so far indicates that both ‘strains’ are present in Australia (Piggott et al. 2021). To date, the majority of *S. frugiperda* damage in Australia has occurred in maize, and no substantial populations have been recorded from turf or pasture.

Understanding the diet breadth, or host range, of invading pestiferous insects is of substantial practical importance. Identifying the host range of a pest species enables pest managers to be informed about which crops are at risk and to determine which plant species may be acting as sources of pest populations. An examination of the host list presented in Montezano et al. (2018) indicates that for many of the host records catalogued there is no substantial evidence to support host plant status, as discussed in Cunningham and Zalucki (2014). To qualify as a true ‘host’ for *S. frugiperda* a plant must be located by gravid female moths in the field, moths must then oviposit and larvae must feed upon it, completing their development to produce reproductive adults. One complicating factor for the host list of *S. frugiperda* is that late-instar larvae exhibit the characteristic armyworm/cutworm behavior of dispersing from grass hosts to infest and damage neighboring crops (Luginbill 1928). Using the aforementioned criteria, records of this nature do not constitute host plant status for the secondarily infested plants, but they may be legitimately considered food plants.

Here we examine the host status of several economically important crop species grown in Australia that are listed as host plants of *S. frugiperda* (Montezano et al. 2018). We investigated oviposition behavior and larval performance of an Australian population of *S. frugiperda* on five crop species: maize (*Zea mays*), sorghum (*Sorghum bicolor*), peanut (*Arachis hypogaea*), cotton (*Gossypium hirsutum*), and pigeon pea (*Cajanus cajan*). All five crops are cultivated throughout Northern Australia, where *S. frugiperda* has recently invaded and established.

## Materials and Methods

### Insects

Moths and larvae used in experiments were derived from a laboratory culture of *S. frugiperda* maintained at the Queensland Department of Agriculture and Fisheries laboratory in Toowoomba, Australia. The culture was initially established from field-collected larvae from maize and sorghum crops throughout Queensland and regularly supplemented with field-collected specimens to minimize inbreeding. The culture was kept in a controlled temperature room maintained at 25°C, 60% humidity, and a 12:12 h photoperiod. Moths were kept in 5-liter plastic buckets and supplied with 10% sucrose solution from 70-ml plastic specimen jars with cotton wicks. An 18-cm hole was cut in the bucket lid and the edges of the lid were used to secure nappy liner (bamboo rayon) which was used as an oviposition substrate along with paper toweling placed over the internal walls of the buckets. Egg masses were removed and stored in polyethylene plastic bags until hatching. Neonate larvae were placed in groups in 650-ml rectangular plastic containers filled with sweet-corn incorporated soybean-flour based artificial diet (see ingredients list; Supp Table S1 [online only]). Upon reaching third instar, larvae were transferred onto fresh diet in 32-well plastic trays where they remained until pupation. Pupae were washed in 1% sodium hypochlorite bleach solution and placed on paper towel in plastic containers (17.5 × 12 × 6 cm) within cages (60 × 60 × 60 cm) until moth emergence.

### Plants

Oviposition and larval performance experiments were conducted on plants of the selected crop cultivars (Table 1). Plants were grown in a temperature-controlled glasshouse (27°C day, 25°C night) under natural photoperiod. Seeds were planted into a mixture of 2:1 Searles Premium potting mix and sand in 4L pots. Plants were watered regularly, as required, and no additional fertilizer was provided. Experiments were conducted on plants approximately 21 d after planting (range: 17–24 d), corresponding to crop stages listed in Table 1. Except for the final two pupation experiments, where older, larger plants were used (27–29 d) to provide more biomass for later instar larvae. None of the cultivars used in experiments were transgenic, nor were any insecticidal treatments applied to seeds or plants.

**Table 1.** Crop species, their cultivars, and crop stages used in the *S. frugiperda* oviposition preference, neonate silking, and larval performance experiments

Crop (species name)	Cultivar (seed source)	Crop stage examined^*^
Maize (*Zea mays*)	PAC 606IT (Pacific Seeds)	V4–V6
Sorghum (*Sorghum bicolor*)	Resolute (Pacific Seeds)	V4–V6
Peanut (*Arachis hypogaea*)	Menzies (Peanut Company of Australia)	V4–V6
Pigeon pea (*Cajanus cajan*)	ICPL 86012 (ICRISAT)	V3–V5
Cotton (*Gossypium h*ir*sutum*)	Sicot 620 (Cotton Seed Distributors)	V3–V4

Cultivars were selected as they are currently grown in Australian farming systems.

^*^V-stage for grasses (maize and sorghum) corresponds to the count of leaves with leaf-collars present, whereas for the broadleaf crops (peanut, pigeon pea, and cotton), V-stage represents the count of nodes on the mainstem.

### No-Choice Oviposition Experiment

Pupae for the oviposition experiment were separated by sex by examination under a stereomicroscope (Nikon, SMZ800N). Male and female pupae were placed in separate emergence cages (60 × 60 × 60 cm) that were checked daily for emerging moths. Upon emergence, moths were removed from cages and placed into groups of 10 (5 males and 5 females) in oviposition cages (68.5 × 68.5 × 121.9 cm) in the glasshouse where they were provided with 10% sucrose solution. Moths were left to mate and oviposit for 4 nights in the cages, which contained three plants of a single crop species (one plant per pot). After 4 nights, we recorded the number, location, and size (length and width) of any egg masses present. For eggs laid on plants, the number of eggs per mass was counted under a stereomicroscope. Four replicates were performed for each crop species.

### Neonate Silking

To obtain a measurement of neonate dispersal off plants by ‘silking’ or ‘ballooning’, behavioral assays were conducted in a laboratory using whole plants of the five crop species. Egg masses laid on nappy liner were collected from the laboratory colony the morning after being laid and based on the degree-days (DD) for egg hatching, maintained at 27°C until they reached the ‘black-head’ stage (ca. 35 DD above 13°C; Du Plessis et al. 2020). All larvae used in this experiment were allowed to hatch and feed on their egg chorion and were used for assays within <1 h of hatching. We artificially infested plants with 10 neonates per plant. After neonates were placed on plants, they were semi-continuously observed by a single observer. Whenever a larva was observed to drop off a plant by a silk thread, it was collected and removed from the experiment. Plants were watched for a period of 2 h with notes recorded every 10 min on how many larvae had silked or remained on plants. At the end of 2 h, plants were dissected to ensure the correct number of larvae remained on plants. Six replicates were conducted, with a replicate constituting a block with one plant of each crop species.

### Early Instar Larval Performance

#### Larval Performance (Survival, Development, and Weight) to 5 d

In order to examine the performance of early instar larvae, the stages most susceptible to mortality (Zalucki et al. 2002), we artificially infested crop plants with egg masses and then measured variables defining larval performance after 5 d. As in the silking experiment, egg masses were obtained from the laboratory colony and maintained at 27°C. Upon reaching the ‘black-head’ stage, egg masses were divided into groups of 50 eggs using a scalpel under a stereomicroscope. Egg masses were then fixed to the underside of the uppermost fully expanded leaf on plants in the glasshouse, by ‘gluing’ the nappy liner to the leaf surface with chicken egg albumen. All egg masses were observed hatching within a few hours after they were placed on plants. Plant pots were placed within saucers and placed in large (180 × 107 cm) galvanized trays placed on glasshouse benches and filled with water (3 cm depth) to form a moat in an attempt to prevent larvae moving between plants. Five days after egg mass placement, plants were destructively harvested and the number, instar, and weight of surviving larvae assessed. Five blocked replicates were performed.

#### Larval Performance (Survival and Development) to 8 d

In our second early instar larval performance experiment, we examined performance of larvae from hatching through to 8 d after egg mass placement. ‘Black-head’ egg masses were divided to a size of *n* = 10 eggs per mass and attached to plants, with one egg mass attached per plant. Plant pots were positioned within plastic saucers and trays as in the previous experiment. After 8 d plants were dissected, and the number of live larvae and their stage of development recorded. We placed surviving larvae on fresh, undamaged plants to examine further larval performance; however, 2 d later (10 d after egg placement), we terminated the experiment as we noticed later instar larvae were dispersing from plants. Therefore, we only analyzed the performance measures (survival and development) recorded 8 d after egg mass placement. Four blocked replicates were performed.

### Later Instar Larval Performance

#### Larval Feeding and Movement

We conducted two experiments examining the performance of later instar *S. frugiperda* larvae, beginning at fourth instar. In both experiments, larvae were kept on artificial diet for their first three larval instars. Newly moulted fourth-instar larvae were starved for 4 h and weighed to obtain initial weights. The setup for our first later instar experiment consisted of two plants (27 d after planting) of the same crop species within a single 4-liter pot. The pot was positioned within a plastic saucer, which was placed inside a larger 18-liter pot. The larger pot was filled with water and detergent to the base of the saucer, enabling the larger pot to be used as a moat to detect larval movement away from plants.

Fourth-instar larvae were placed on plants either 1) in the whorl of the monocot crops or 2) the uppermost fully expanded leaf of the broadleaf crops. Plants were checked daily for the presence of larvae (which were often difficult to detect due to their concealed feeding habit), any signs of feeding damage on plants, and the moats were checked for drowned larvae. One fourth-instar larva was placed per pot (i.e., two plants) and each crop species was replicated fourteen times (i.e., 14 pots). Seven days after placement, surviving larvae were removed from plants, taken to the laboratory, weighed, and their stage of development was recorded.

#### Relative Growth Rate and Development to Pupae

In the final experiment, recently moulted fourth-instar larvae were restricted to 29-d-old plants with the use of mesh bags (30 × 15 cm). For the broadleaf crops, this was done by covering the main stem of plants. However, for maize and sorghum, expanded leaves had to be bunched together to fit leaves (and most importantly, the plant whorl) into the bag. Bags were tied around the plant stem to prevent larval escape. Seven days after placement, the bags were removed, and plants transferred to the laboratory. Larval instar and weight were recorded 7 d after placement, and larvae were then returned to new undamaged plants in the glasshouse, where they remained for another 7 d. Relative growth rates (RGR) at 7 d were calculated for each larva using the following formula:


$$\text{RGR}=\left(ln\left({\text{wt}}_{1}\right)-ln\left({\text{wt}}_{0}\right)\right)/\left(t_{1}-t_{0}\right),$$

where wt_1_ and wt_0_ represent larval weights (in mg) at the sample point (i.e., 7 d after placement) and their initial weight, respectively, and t_0_ and t_1_ represent sample 1 and 0 times (i.e., days after larval placement, and placement day).

At 14 d after placement, bags were removed and insects were transferred to the laboratory and the stage of development (larval instar, pupa) recorded. Ten replicates were performed for maize, sorghum, pigeon pea, and cotton, but due to availability of plants, only 6 replicates were performed for peanut. During the experiment, two larvae were able to make their way out of the bags on peanut plants, and a single larva was able to escape from a bag on a maize plant.

### Statistical Analysis

One-way ANOVA was used to analyze the oviposition experiment, larval silking experiment, and most measures (weights and proportions) recorded in larval performance experiments. For all ANOVAs, post-hoc multiple comparisons were performed using Fishers-LSD test in the R package ‘agricolae’ (De Mendiburu 2020). Kruskal–Wallis rank sum test was used to compare the total number of eggs laid on plants among crop varieties in the oviposition experiment, because data were not normally distributed even after transformation. Replicate was used as a blocking factor in the silking experiment along with the first and second larval performance experiments. Pearson’s chi-square test was used to analyze the frequency of larval survival, pupation, and plant damage in the two final larval performance experiments. All statistical analysis was performed in R version 3.6.2 (R Core Team 2019).

## Results

### Oviposition No-Choice Experiment

**Fig. 1.**
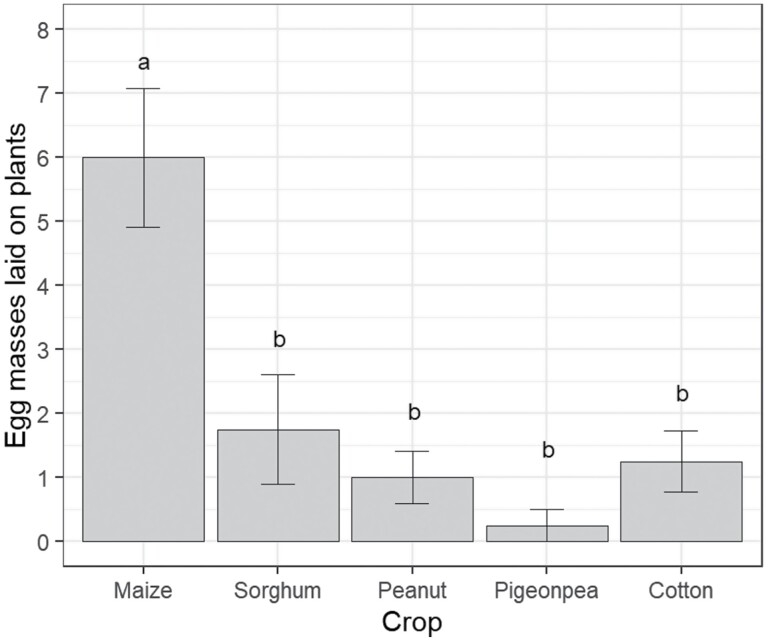
Number of egg masses laid on plants by *S. frugiperda* in the oviposition no-choice experiment. Bars indicate means and error bars are standard error of the means. Different letters above bars indicate a significant difference among crop species according to Fishers-LSD test.

*Spodoptera frugiperda* moths laid eggs on all five crop species; however, moths laid more egg masses on maize than any other crop (*F* = 10.98; df = 4, 15; *P* < 0.001; Fig. 1). The total number of eggs laid on plants was highly variable, ranging from 0 to 1509 eggs in a single cage, and differed only marginally among crop species (χ^2^ = 9.51; df = 4; *P* = 0.049), with the most eggs (mean = 748.8 ± 221) laid on maize. The number of eggs per mass varied between 5 and 675 (mean = 135.6 ± 20) but did not differ among crops (*F* = 0.59; df = 4, 9; *P* = 0.68). Large numbers of egg masses were laid on surfaces other than plants (the cage wall or adult diet fountain; Supp Fig. S1 [online only]); however, this did not differ among crop species (*F* = 0.22; df = 4,15; *P* = 0.92). Nor was there any difference among the total number of egg masses laid (masses laid on plants plus other surfaces; *F* = 2.33; df = 4, 15; *P* = 0.10).

### Neonate Silking Experiment

**Fig. 2.**
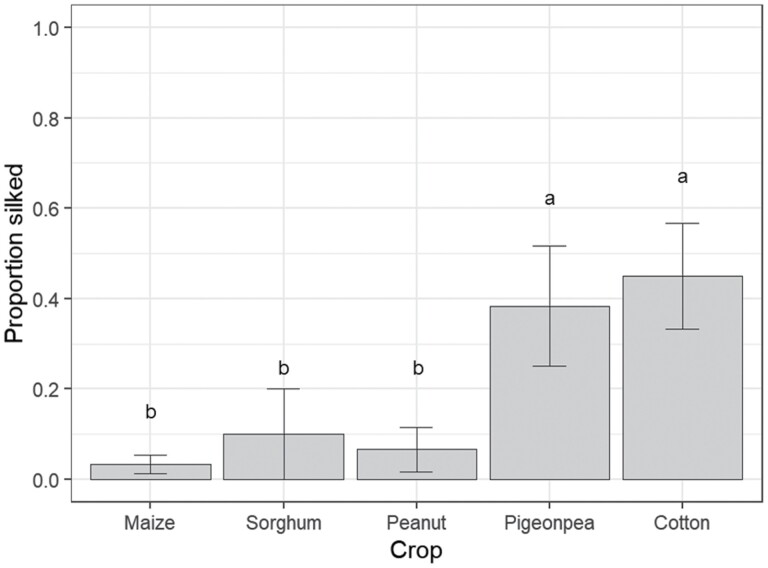
Proportion of *S. frugiperda* neonates silking from plants in the neonate silking experiment. Bars indicate means and error bars are standard error of the means. Different letters above bars indicate a significant difference among crop species according to Fishers-LSD test.

The proportion of larvae silking differed among crop species (*F* = 4.29; df = 4, 20; *P* = 0.012; Fig. 2). Greater proportions of larvae dispersed by silking within 2 h on cotton (45%) and pigeon pea (38%) than on maize, peanut, or sorghum plants (Fig. 2).

### Early Instar Larval Performance

#### Larval Performance (Survival, Development, and Weight) to 5 d

**Fig. 3.**
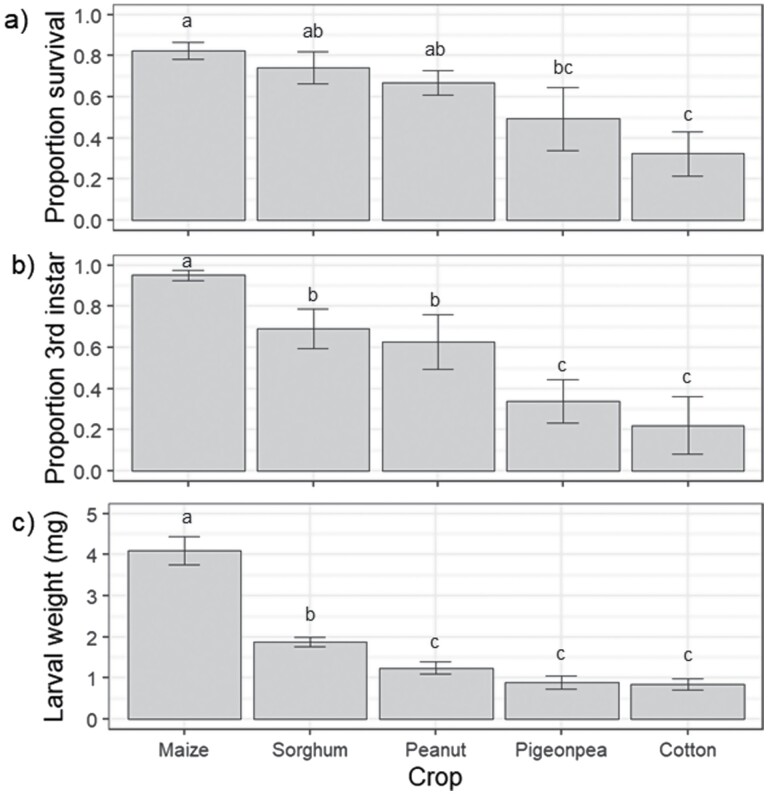
Larval performance measures recorded 5 d after egg mass placement (*n* = 50 eggs per mass) in the early instar performance experiment: (a) larval survival, (b) larval development, and (c) larval weight. Bars indicate means and error bars are standard error of the means. Different letters above bars indicate a significant difference among crop species according to Fishers-LSD test.

Crop species affected all larval performance variables measured 5 d after egg mass placement (Fig. 3). Survival significantly differed among crops (*F* = 3.92; df = 4, 16; *P* = 0.021), with the most larvae (82%) surviving on maize and the fewest (32%) on cotton. Larval development was also affected by crop species (*F* = 12.41; df = 4, 16; *P* < 0.001), again development was fastest on maize with 95% of larvae reaching third instar within 5 d, whereas on cotton only 22% of larvae developed to this stage by this time. Finally larval weight significantly differed among crops (*F* = 48.53; df = 4, 16; *P* < 0.001), larvae reared on maize weighed over twice as much as the second-ranked sorghum and approximately four times as much as larvae feeding on peanut, pigeon pea, and cotton (Fig. 3).

#### Larval Performance (Survival and Development) to 8 d

**Fig. 4.**
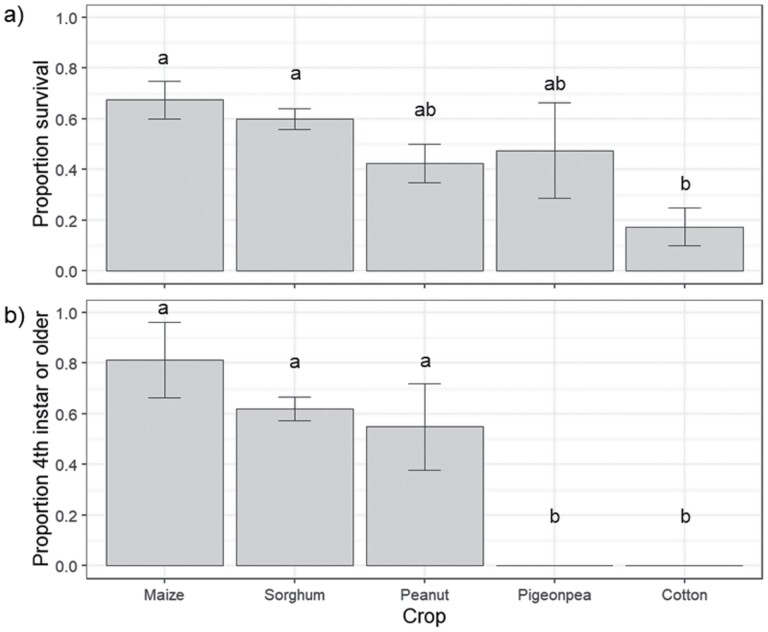
Larval performance measures recorded 8 d after egg mass placement (*n* = 10 eggs per mass) in the second early instar performance experiment: (a) larval survival and (b) larval development. Bars indicate that means and error bars are standard error of the means. Different letters above bars indicate a significant difference among crop species according to Fishers-LSD test.

At 8 d after egg mass placement, there was a significant effect of crop species on larval survival (*F* = 3.64; df = 4, 12; *P* = 0.037; Fig. 4). Maize ranked highest for survival (67.5%) and cotton ranked lowest (17.5%). Using the proportion of larvae that had developed to fourth instar or greater as a proxy measure, larval development rate differed among crop species (*F* = 12.5; df = 4, 12; *P* < 0.001; Fig. 4).  By 8 d after egg mass placement, over half of all surviving larvae had reached fourth instar on maize, sorghum, and peanut, with a single larva on maize even reaching the fifth instar (Supp Fig. S2 [online only]). Most larvae feeding on pigeon pea were third instars (63%), whereas most larvae feeding on cotton (77%) remained in the second instar.

### Later Instar Larval Performance

#### Larval Feeding and Movement

Within 48 h of placement, all larvae placed on cotton and pigeon pea were either dead in the moat or had disappeared and were not recovered. Whether or not plants suffered visible feeding damage also differed among crop species (χ^2^ = 36.19; df = 4; *P* < 0.001). All maize and sorghum plants had visible feeding damage but for peanut, pigeon pea, and cotton, 2/14, 11/14, and 9/14 replicates, respectively, showed no evidence of larval feeding. By 7 d after placement, a substantial proportion (89%) of larvae had dispersed off plants and were either located dead in the moat or not recovered. The number of larvae remaining on plants 7 d after placement differed among crop species (χ^2^ = 13.55; df = 4; *P* = 0.0089). Of the 8 larvae remaining on plants after 7 d, half were on sorghum and half were on maize. Of these live larvae, seven were fifth instars, and one was a sixth instar (a sorghum replicate).

#### Relative Growth Rate and Development to Pupae

**Fig. 5.**
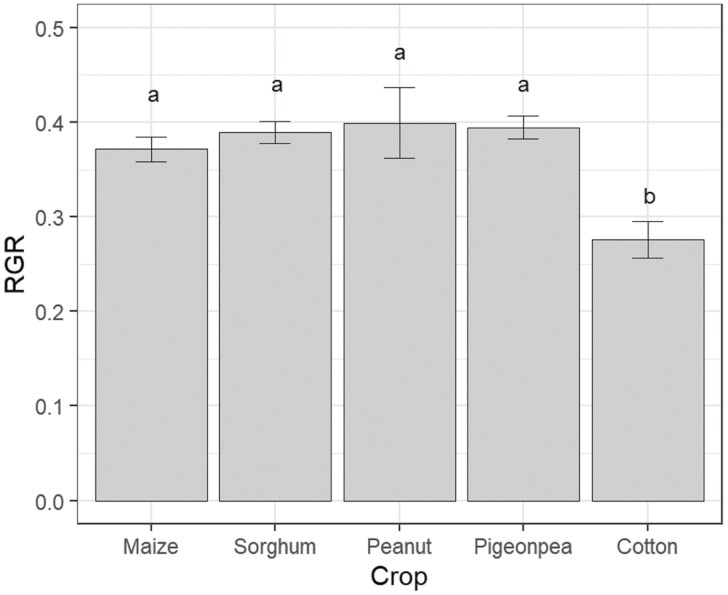
Relative growth rate of larvae recorded 7 d after placement in the second late instar larval performance experiment. Bars indicate means and error bars are standard error of the means. Different letters above bars indicate a significant difference among crop species according to Fishers-LSD test.

At 7 d after initial placement, larval relative growth rate differed among crop species (*F* = 8.96; df = 4, 40; *P* < 0.001). Larvae on cotton had a substantially reduced relative growth rate in comparison with the other four crop species (Fig. 5). At 7 d after placement, there was a significant effect of crop species on development (χ^2^ = 18.91; df = 8; *P* = 0.015), with larvae developing best on sorghum (50% of larvae had reached sixth instar).

At 14 d after the initial placement, larvae had pupated on all crop species. There was a difference in the number of larvae that had pupated among crop species (χ^2^ = 19.50; df = 4; *P* < 0.001), with the most pupating on sorghum (100%) and the fewest on cotton (30%). The seven larvae that had not pupated on cotton were still live sixth instars.

## Discussion

Female *S. frugiperda* moths laid readily on maize, with more egg masses on maize than other crop plants. The preference of *S. frugiperda* to oviposit on maize has been reported previously (Guo et al. 2021, He et al. 2021a, Sotelo-Cardona et al. 2021) and moths are attracted to volatiles of undamaged maize plants (Signoretti et al. 2012). However, this preference for maize may vary according to *S. frugiperda* strain (Meagher et al. 2011). Ovipositional preference of *S. frugiperda* populations may be relatively plastic, with a shift of preference from maize to rice occurring within four generations of continuous feeding on rice (Hafeez et al. 2021). Although *S. frugiperda* did lay egg masses on the four other crops in our experiments, they laid many more eggs on the cage wall in the no-choice tests with these plants (Supp Fig S1 [online only]). Large levels of oviposition on cage walls have been recorded in other experiments (Guo et al. 2021, Sotelo-Cardona et al. 2021). This may be partially explained by the responses of *S. frugiperda* females to tactile cues and a preference for ‘grooved’ surfaces (Rojas et al. 2003).

Pigeon pea and cotton experienced the highest levels of neonate larvae silking in the laboratory experiment. Neonates may encounter cues on pigeon pea and cotton that encourage them to disperse away from the plant either in search of a better feeding site or another plant species (Zalucki et al. 2002). Interestingly, even though moths only had a strong oviposition preference for maize, a large proportion of neonate larvae remained and fed on both sorghum and peanut in addition to maize. These silking data correlate with our early instar performance results, as most neonate larvae remained on the three highest ranked crop species in our early instar performance experiments (maize, sorghum, and peanut), whereas survival and growth were poor on pigeon pea and cotton.

The larval survival presented in the performance experiment represents the remaining larvae, excluding those that either left the plant by silking or died due to plant effects (i.e., plant defences). The survival of early instar larvae was best on maize, again followed by sorghum and peanut, and least on pigeon pea and then cotton. Maize was again the best plant for larval development and growth. Peanut and sorghum grouped together for development rate, although larvae on sorghum were twice the weight of those feeding on peanut. Again, pigeon pea and cotton were the lowest ranked plants for development and larval size. When larval performance was examined up to 8 d, once again larval survival and development was best on maize and lowest on cotton.

Our performance experiments using fourth-instar larvae found that large larvae have a strong drive to disperse away from non-preferred plant species (i.e., cotton and pigeon pea), likely in search of a more suitable food plant. However, the caged experiment indicated that if larvae are restricted to these plants, they are indeed able to develop to pupae. This dispersal behavior of later-instar larvae is poorly understood, and we believe that its significance is underappreciated. The observation that larvae eventually dispersed from even preferred plant species (maize and sorghum) indicate that there are triggers (likely from both the plant and the larvae) that initiate this dispersal behavior. We expect that better understanding of these triggers will help guide management of *S. frugiperda*, particularly during its armyworm/cutworm foraging mode, and accordingly we plan to examine this behavior in future experiments.

Are the five crop species examined in our study host plants of *S. frugiperda*? In a series of simple experiments, we have identified some major differences among the crops that we examined in terms of *S. frugiperda* oviposition behavior, larval dispersal, and larval performance. Our data indicate that maize is highly preferred by *S. frugiperda* and that larvae perform well on it in comparison with the other crops; this is consistent with historical field evidence that maize is a highly preferred host plant of *S. frugiperda* (Luginbill 1928, Buntin 1986, Day et al. 2017, Overton et al. 2021). Sorghum, like maize, has long been recorded as a host plant that suffers economic damage from *S. frugiperda* (Luginbill 1928, Buntin 1986). However, sorghum appears to be at less risk across the invasion front than maize (Fotso Kuate et al. 2019, Hailu et al. 2021, Niassy et al. 2021). In Queensland, sorghum is the major summer grain crop cultivated with an annual mean production area of 372,000 ha compared with only 31,000 ha for maize (ABARES, 2022). Yet very few damaging infestations have been recorded from sorghum, but damaging populations are consistently reported from maize. These observations align with our results which suggest that sorghum is not preferred for oviposition and that early instar larvae do not perform well on sorghum plants compared with maize.

Consistent with other recent work (He et al. 2021a), our results show that peanut is not preferred by *S. frugiperda* for oviposition but that larvae can feed on and survive on this crop (Deitz et al. 1992). Larvae perform better on peanut than on pigeon pea or cotton, and for some metrics (neonate silking, survival, and development), they perform as well on this crop as they do on sorghum. *Spodoptera frugiperda* is a sporadic pest of cotton in the Americas that sometimes warrants control measures (Hardke et al. 2015). However, studies, in addition to ours, show larvae lack a feeding preference for cotton and perform poorly when restricted to feeding on cotton plants (Ali et al. 1990a, Ali et al. 1990b, Silva et al. 2017). Finally, we were unable to find any published evidence to support the claim that pigeon pea is a host of *S. frugiperda*. As *S. frugiperda* has made its way across south Asia, where pigeon pea production is significant, there are few reports of it damaging pigeon pea crops. Indeed, pigeon pea is often used as an intercrop for maize in Africa (Kuyah et al. 2021) and may even repel *S. frugiperda* moths as part of a push-pull system (Midega et al. 2018). Our results combined with the published literature indicate that maize and sorghum can be considered host plants for the *S. frugiperda* population we examined, whereas peanut, cotton, and pigeon pea should be classed as ‘food plants’ but not hosts according to our criteria and available evidence. However, these results may differ based on the geographic location of *S. frugiperda* populations, the phenological stage of the plant available, and the environmental context.

We recommend the two ‘feeding modes’ in which *S. frugiperda* is a pest of crops should be more explicitly distinguished. Firstly, the ‘defoliator’ whereby moths enter a crop and lay eggs, larvae then hatch and feed on the crop throughout their development. The second feeding mode has been referred to as the ‘armyworm’ or ‘cutworm’ mode whereby eggs are typically laid on grasses (usually crops or agricultural weeds) and older instar larvae transition from feeding on these grass hosts to a nearby crop (either because they have exhausted their food supply or it has been desiccated; Luginbill 1928). We suspect that many of the host plants listed for *S. frugiperda* (Montezano et al. 2018) are records of this nature. For instance, since *S. frugiperda* has entered Australia, there are similar records where large *S. frugiperda* larvae have been recorded in soybeans, sugarcane, and even ornamental *Heliconias.*

Environmental context likely plays an important role in which plant species are attacked by *S. frugiperda*—in both the ‘armyworm’ and ‘defoliator’ feeding modes. The apparency of a plant species and the *S. frugiperda* population present in a landscape likely interact to determine which of the available plant species are used by *S. frugiperda*. For instance, if there is a large *S. frugiperda* population present in the landscape and there is an abundance of a non-preferred plant species, there may well be oviposition on the plants and ultimately damaging larval populations on what is typically a non-preferred plant. Scenarios like this may explain damaging infestations of *S. frugiperda* in broadleaf crops (e.g., cotton and soybean) in Southern United States (Nagoshi et al. 2007b). We suggest that in future, records of *S. frugiperda* infestations should include 1) whether the *S. frugiperda* larval population was a result of oviposition on the plants (i.e., defoliator), or from large larvae moving from a grass host onto the plants (i.e., armyworm), and 2) the environmental context—was there a large *S. frugiperda* population in the landscape, high oviposition pressure, or an abundance of surrounding host plants (e.g., maize)?

As *S. frugiperda* has spread across the globe, the extensive list of host plants has caused considerable anxiety and distress for farmers. Our results indicate that host plant records should be evaluated more critically. Many studies of a similar nature to ours rely on experiments using excised plant parts in the laboratory. However, the relevance of these results is often questionable. For instance, Sotelo-Cardona et al. (2021) found similar performance of larvae on maize, cabbage, soybean, and tomato based on measures of mortality and pupal weight when insects were confined to excised leaves in sealed containers. Yet to date, reports of soybean, tomato or cabbage being attacked by *S. frugiperda* across its invasion front are rare while maize is commonly attacked, even though the former crops are present. Similarly, in our caged-larvae experiment, we found similar performance of older instar larvae on all tested crops except cotton. We expect that if larvae were able to disperse to undamaged plants (as in a field scenario), later instar performance may have been different, as indicated by the substantial difference among larval performance in the early instar experiments. Larvae have a strong preference for feeding within the whorl of maize and sorghum, and we expect larvae may leave to feed on an undamaged plant and likely feed on several plants throughout their development during early vegetative crop stages.

We question the importance and relevance of experiments that restrict larvae onto excised plant parts in Petri dishes or other small containers in the laboratory. Indeed, our and other data indicate that although *S. frugiperda* larvae do feed on a wide variety of plant species, including many crops, many of these crops are neither ‘preferred’ by adults for oviposition nor by immatures for feeding. If the immature stages of an insect are confined to plants or excised leaves and a proportion develops to pupate, does that make the plant species a ‘host plant’ in the field? Similarly, if a later instar larva has moved from a grass to a neighbouring crop, as regularly occurs, should the secondarily infested plant be classed as a ‘host plant’ or more conservatively as a ‘food plant’? Future lists assessing host-range should discriminate between records of this nature and provide more information to better inform which crop species may be at risk. Despite all five crops examined in our study being listed as host plants (Montezano et al. 2018), since *S. frugiperda* has invaded Australia damaging larval infestations have only been recorded from maize, sorghum, and peanut.

For globe-trotting pestiferous insects, such as *S. frugiperda*, a worldwide collaborative research effort is required to understand the host-range and identify which crops are at greatest risk. An understanding of pest diet breadth can be gained by adopting appropriate methods to examine the preference of moths and performance of larvae on whole plants. Future research comparing host use of *S. frugiperda* across its geographic range using such methods will likely prove very useful. Determining the host-range of invasive insects is vitally important, and this case study of *S. frugiperda* may be used to guide research on incursive agricultural pests in the future.

## Supplementary Material

toac056_suppl_Supplementary_Figure_S1

toac056_suppl_Supplementary_Figure_S2

toac056_suppl_Supplementary_Table_S1
