# Supplementary figures and images for: What Defines a Host? Oviposition Behavior and Larval Performance of Spodoptera frugiperda (Lepidoptera: Noctuidae) on Five Putative Host Plants
Source: J Econ Entomol. 2022 Dec 14;115(6):1744–51. doi: 10.1093/jee/toac056 (PMC9748544; doi:10.1093/jee/toac056)

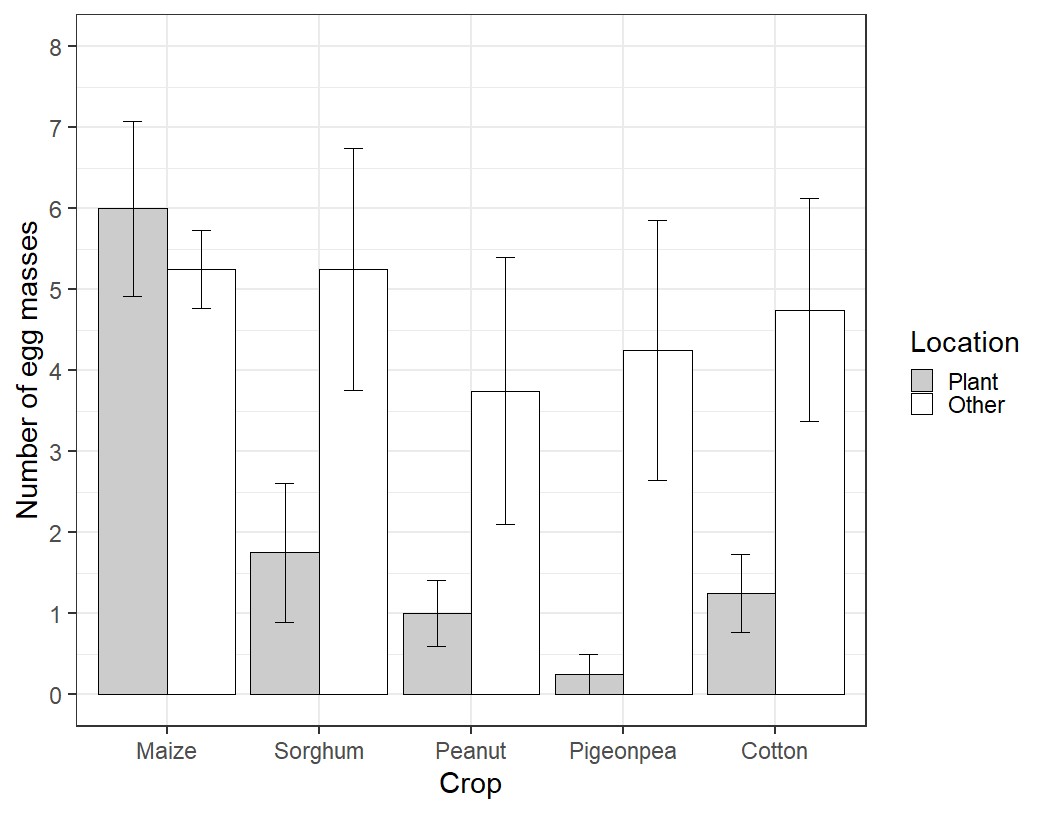

Supplement: toac056_suppl_Supplementary_Figure_S1 [file toac056_suppl_supplementary_figure_s1.jpeg]

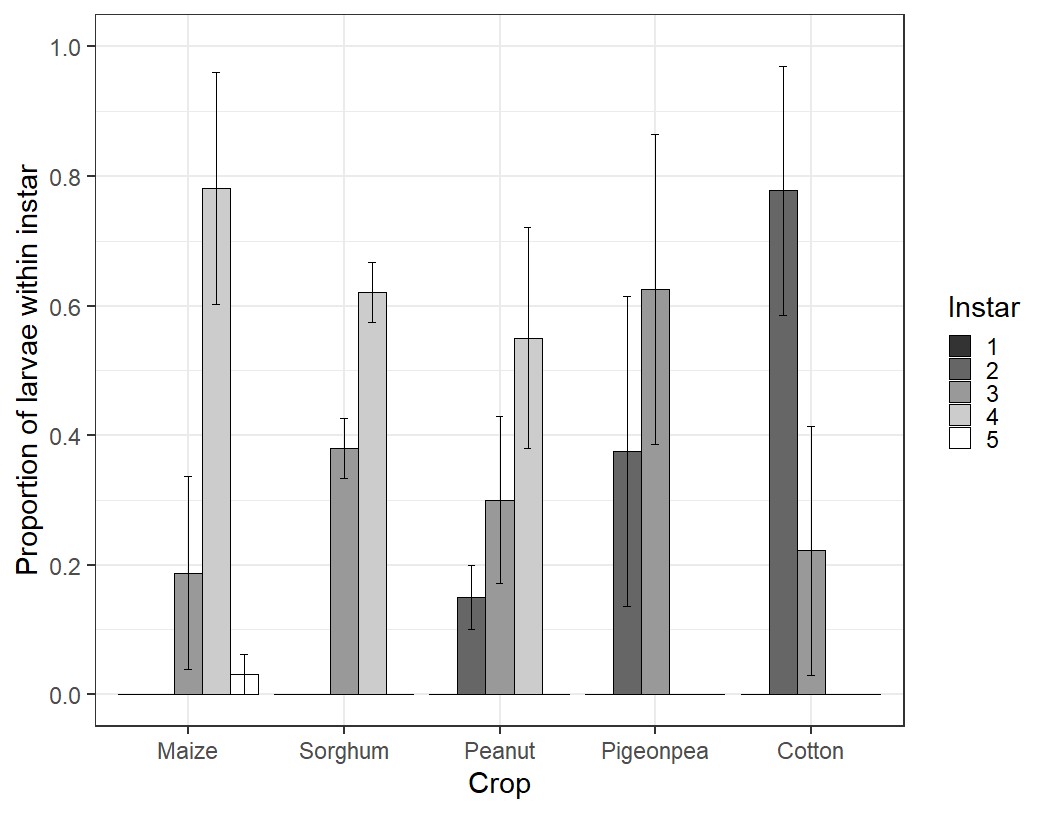

Supplement: toac056_suppl_Supplementary_Figure_S2 [file toac056_suppl_supplementary_figure_s2.jpeg]
