# Supplementary material for: What Defines a Host? Oviposition Behavior and Larval Performance of Spodoptera frugiperda (Lepidoptera: Noctuidae) on Five Putative Host Plants
Source: J Econ Entomol. 2022 Dec 14;115(6):1744–51. doi: 10.1093/jee/toac056 (PMC9748544; doi:10.1093/jee/toac056)
Supplement: toac056_suppl_Supplementary_Table_S1 [file toac056_suppl_supplementary_table_s1.docx]

**Table S1:** Ingredients for artificial *Spodoptera frugiperda* diet used in maintaining the laboratory culture.

| **Ingredient** | **Amount** |
| --- | --- |
| Agar | 50g |
| Distilled water | 3325mL |
| Soybean flour | 340g |
| Raw wheat germ | 240g |
| Brewer’s yeast | 200g |
| L-Ascorbic acid | 12g |
| Nipagin | 12g |
| Sorbic acid | 4g |
| Sweet corn | 150g |
| Mould inhibitor  (Propionic acid, Phosphoric acid, Distilled water (7:1:9)) | 11mL |
